# Genetic polymorphisms of lncRNA-p53 regulatory network genes are associated with concurrent chemoradiotherapy toxicities and efficacy in nasopharyngeal carcinoma patients

**DOI:** 10.1038/s41598-017-08890-2

**Published:** 2017-08-16

**Authors:** Youhong Wang, Zhen Guo, Yu Zhao, Yi Jin, Liang An, Bin Wu, Zhaoqian Liu, Xiaoping Chen, Xiang Chen, Honghao Zhou, Hui Wang, Wei Zhang

**Affiliations:** 1 Department of Clinical Pharmacology, Xiangya Hospital, Central South University and Institute of Clinical Pharmacology, Central South University; Hunan Key Laboratory of Pharmacogenetics, Changsha, 410008 P.R. China; 2 Department of Radiation Oncology, Hunan Provincial Tumor Hospital & Affiliated Tumor Hospital of Xiangya Medical School, Central South University; Hunan Key Laboratory of Translational Radiation Oncology, ChangSha, 410013 P.R. China; 3 Department of Dermatology, Xiangya Hospital, Central South University; Hunan Key Laboratory of Skin Cancer and Psoriasis, Changsha, Hunan 410008 China

## Abstract

The relevance of the transcription factor p53 in cancer is inarguable, and numerous lncRNAs are involved in the p53 regulatory network as either regulators or effectors, triggering a transcriptional response that causes either cell arrest or apoptosis following DNA damage in a p53-dependent manner. Despite the fact that the therapeutic response is improved in NPC, heterogeneity among people remains with regard to the susceptibility of adverse effects and the efficacy of treatments. Therefore, we analysed eight potentially functional SNPs of five genes in the lncRNA-p53 regulatory network in a discovery cohort of 505 NPC patients. By performing multivariate logistic regression, the impact of genetic variations on the efficacy and risk of CRT-induced toxicities was investigated. The most dramatic finding was that the *MEG3* rs10132552 CC genotype had a greater than three-fold increased risk of developing grade 3–4 anaemia (OR = 3.001, 95%CI = 1.355–6.646, *P* = 0.007). Furthermore, the rs10132552 CT genotype had a better response to treatment (OR = 0.261, 95%CI = 0.089–0.770, *P* = 0.015). Individuals carrying *LINC-ROR* rs2027701 with one or two variant alleles had significant associations with a reduced risk of neutropaenia (OR = 0.503, 95%CI = 0.303–0.835, *P* = 0.008). In conclusion, our results suggested that genetic polymorphisms of the lncRNA-p53 regulatory network could play a potential role in reducing treatment-related toxicities and improving outcomes for NPC patients.

## Introduction

Nasopharyngeal carcinoma (NPC) is an epithelial malignancy with extremely skewed ethnic and geographic distributions and a particularly high prevalence in southern China^1^. Concurrent chemoradiotherapy (CRT), an important therapeutic milestone, is the standard treatment for locally advanced NPC. Although overall survival has been dramatically improved by the advancement of radiotherapy technology as well as the broader application of chemotherapy, CRT-induced acute toxicity remains a challenge, as it is multifactorial and difficult to predict^2^. Chemoradiotherapy is invariably associated with higher incidences of haematological and non-haematological acute toxic effects compared with radiotherapy alone^3–5^. There is significant variation in prognosis and the risk of toxicities among patients, even if they are exposed to the same therapeutic regimens, suggesting that genetic polymorphisms play a crucial role in individual susceptibility to toxicities and sensitivity to treatments^6,7^.

In support of this notion, many studies have illustrated that single nucleotide polymorphisms (SNPs) may be useful as independent factors for predicting the toxicities and curative efficacy of chemoradiotherapy in human cancers including NPC. For example, the rs1982073 polymorphism of *TGFB1* seems to trend with a higher risk of mucositis in head and neck squamous cell carcinoma when patients underwent platinum-based CRT^8^. As indicated by Ming Jia^9^, the *GADD45B* rs2024144T variant allele correlated with a major event in response to severe haematologic toxicities in individuals with non-small cell lung cancer. Similarly, SNPs in DNA repair pathway genes were correlated with sensitivity to radiotherapy and chemotherapy^10^.

**Figure 1:**
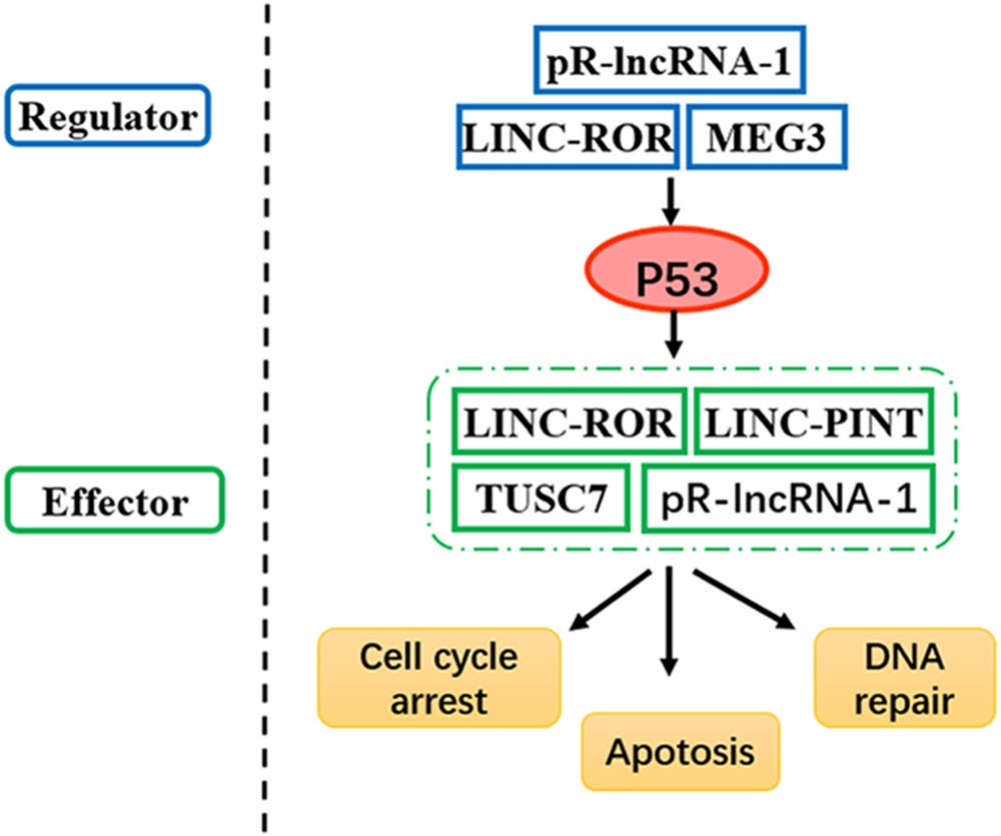
LncRNAs serve as p53 regulators and effectors, participating in the p53 regulatory pathway. On the one hand, lncRNAs are able to regulate p53 directly or indirectly at the transcriptional or posttranscriptional levels such as *MEG3*. On the other hand, several lncRNAs could be induced or suppressed by p53 such as *LINC-ROR, pR-lncRNA-1, LINC-PINT* and *TUSC7*.

LncRNA is a type of long non-coding RNA with transcripts >200 nt in length that functioning as a master regulator controlling protein-coding and non-coding genes at multiple levels; lncRNAs could drive important cancer phenotypes and serve as a biomarker in diverse cancers such as NPC^11–14^. Undoubtedly, p53, a master human tumour suppressor, participates in all steps of tumour initiation and development by regulating the expression of many downstream genes, whose dysfunction is closely related to the occurrence and progression of NPC^15^. LncRNA has now been added to the p53 regulatory pathway **(**Fig. 1 **)** to form a sophisticated regulatory network^16,17^ that has generated increased attention for its potential to contribute to disease.

On one hand, lncRNAs act as p53 effectors that are regulated by p53 directly and modulate gene expression downstream of p53. For example, *LINC-ROR*, *pR-lncRNA-1*, *LINC-PINT* and *TUSC7* alter the interaction between p53 and potential p53 response elements (p53REs) when confronted with cellular stresses such as the DNA damage induced by radiation and/or chemotherapeutics by regulating cell proliferation, cell cycle and cell apoptosis^18–21^. On the other hand, lncRNAs such as *MEG3* can serve as p53 regulators by controlling p53 stability^17^. Based on these examples, we selected eight potential SNPs in five genes *MEG3* (rs10132552T > C)*, LINC-ROR* (rs2027701A > G), *pR-lncRNA-1* (rs73594404G > A and rs3743773G > A), *LINC-PINT* (rs1059698A > C and rs2293750T > A) and *TUSC7* (rs1829346C > A and rs36080650T > C) to determine whether genetic polymorphisms of lncRNA-p53 regulatory network genes are associated with toxicities or the therapeutic efficacy of concurrent chemoradiotherapy in NPC in hopes of discovering valuable new biomarkers for personalized CRT among NPC patients.

## Results

### Patient Clinical Characteristics and Genotype Distribution

The studied cohort included 374 males and 131 females, with a mean age of 47.41 (ranging from 15 to 73) years old. Among these patients, 455 individuals (90.1%) were diagnosed at late stages (III and IV), and the other patients (9.9%) were at early stages (I and II). All of the patients were treated with IMRT, and the median total radiation dose was 71.34 Gy. Regarding chemotherapy, regimes of platinum-based inducing and concurrent chemotherapy were given to participants. The demographic characteristics of NPC patients are described in Table 1. Although none of the patients in this study suffered death caused by toxicities, 51 (10.1%), 129 (25.5%), 121 (24.0%), 73 (14.5%) and 94 (18.6%) experienced grade 3–4 dermatitis, mucositis, myelosuppression, leukopaenia and neutropaenia, respectively. Furthermore, 25 (6.0%) and 62 (15.0%) experienced worse treatment efficacy of CRT at the primary tumour and lymph node, respectively, months after treatments.

**Table 1:** Patient demographics and clinical characteristics.

Patient characteristics	N = 505(%)
**Gender**	
Male	374 (74.1)
Female	131 (25.9)
**Age, years**	
Mean ± SD	47.41 ± 9.15
<47	229 (45.3)
≥47	276 (54.7)
**BMI**	
<18.5	30 (5.9)
18.5 ~ 24	274 (54.3)
≥24	201 (39.8)
**Smoking status**	
Smoker	247 (48.9)
Nonsmoker	258 (51.1)
**Drinking status**	
Drinker	90 (17.8)
Nondrinker	415 (82.2)
**Histological type**	
WHO type II	214 (42.4)
WHO type III	291 (57.6)
**Clinical stage** ^**a**^	
I–II	50 (9.9)
III–IV	455 (90.1)
**T-staging**	
T1–T2	246 (48.7)
T3–T4	259 (51.3)
**N-staging**	
N0–N1	93 (18.4)
N2–N3	412 (81.6)
**Grade 3–4 toxic reactions**	
Dermatitis	51 (10.1%)
Mucositis	129 (25.5%)
Leukopenia	73 (14.5%)
Neutropenia	94 (18.6%)
Myelosuppression	121 (24.0%)
**IC regimen**	
DP	200 (39.6)
FP	92 (18.2)
TP	203 (40.2)
GP	10 (2)
**CRT regimen**	
FP	85 (16.8)
TP	108 (21.4)
DDP	83 (16.4)
NDP	172 (34.1)
DP	57 (11.3)
**pGTVnx**	
Mean ± SD	71.34 ± 2.79
<71.00 Gy	261 (51.7)
≥71.00 Gy	234 (48.3)

Abbreviations: BMI, Body Mass Index; IC regimen, Induction chemotherapy regimens; CRT regimen, concurrent chemoradiotherapy regimen; pGTVnx, irradiation dose.

The characteristics of the 8 SNPs are shown in Table 2. The allelice frequencies of the enrolled SNPs all fit Hardy-Weinberg equilibrium (*P* > 0.05).

**Table 2:** Genotype distribution and MAF of 8 SNPs in *LINC-ROR*, *pR-lncRNA-1*, *LINC-PINT*, *MEG3* and *TUSC7*.

Gene	SNP	SNP Location	Alleles	Genotype Distribution^a^	HWE	MAF	Detectable Rate (%)
LINC-ROR	rs2027701	chr18:54724945-54725445	A/G	174/247/81	0.670	0.3810	99.4
pR-lncRNA-1	rs73594404	chr16:53079364-53079864	G/A	453/50/2	0.625	0.0714	100
	rs3743773	chr16:53077757-53078257	G/A	313/167/25	0.656	0.2476	100
LINC-PINT	rs1059698	chr7:130628844-130629344	A/C	223/222/60	0.677	0.3238	100
	rs2293750	chr7:130629522-130630022	T/A	155/252/96	0.720	0.4143	99.6
MEG3	rs10132552	chr14:101300762-101301262	T/C	251/208/37	0.496	0.2952	98.2
TUSC7	rs1829346	chr3:116428657-116429157	C/A	240/219/46	0.694	0.3476	100
	rs36080650	chr3:116431337-116431837	T/C	241/219/45	0.635	0.3571	100

^a^In the order of wild homozygote/heterozygote/mutant homozygote.

Abbreviations: HWE, Hardy-Weinberg equilibrium; MAF, minor allele frequency (Southern Han Chinese).

### *TUSC7* SNP and the Risk of CRT-induced Dermatitis

We demonstrated that *TUSC7* rs1829346 and rs36080650 were significantly associated with dermatitis **(**Table 3 **)**. Patients carrying the AA genotype of rs1829346 were less resistant to grade 3–4 CRT-induced dermatitis (OR = 2.641, 95%CI = 1.118–6.243, *P* = 0.027). Similarly, the rs36080650 CC genotype was also associated with a prominently higher risk of dermatitis than the CT/TT genotypes (OR = 2.700, 95%CI = 1.141–6.386, *P* = 0.024), which was supported by the recessive model (CC vs CT + TT, OR = 2.544, 95%CI = 1.143–5.662, *P* = 0.022). However, no significant corrections between the risk of oral mucositis and SNPs were found.

**Table 3:** Associations between genotypes and concurrent chemoradiotherapy-induced toxicities

Toxic reactions	SNP	Genotypes	Toxicity grade		OR (95% CI)	*P* ^*a*^
**Dermatitis**			Grade ≤ 2 N (%)	Grade > 2 N (%)		
	**rs1829346**	CC	219 (48.2)	21 (41.2)	1.00 (reference)	
		CA	198 (43.6)	21 (41.2)	1.127 (0.597-2.130)	0.712
		AA	37 (8.1)	9 (17.6)	2.641 (1.118–6.243)	**0.027**
		AA + CA vs CC			1.407 (0.767–2.584)	0.270
		AA vs CA + CC			2.492 (1.121–5.540)	**0.025**
	**rs36080650**	TT	220 (48.5)	21 (41.2)	1.00 (reference)	
		CT	198 (43.6)	21 (41.2)	1.130 (0.598–2.135)	0.706
		CC	36 (7.9)	9 (17.6)	2.700 (1.141–6.386)	**0.024**
		CC + CT vs TT			1.414 (0.770–2.595)	0.264
		CC vs CT + TT			2.544 (1.143–5.662)	**0.022**
**Neutropenia**			Grade ≤ 2 N (%)	Grade > 2 N (%)		
	**rs2027701**	AA	134 (32.6)	40 (42.6)	1.00 (reference)	
		GA	205 (49.9)	42 (44.7)	0.545 (0.319–0.930)	**0.026**
		GG	70 (17.0)	11 (11.7)	0.378 (0.171–0.836)	**0.016**
		GG + GA vs AA			0.503 (0.303–0.835)	**0.008**
		GG vs GA + AA			0.550 (0.264–1.146)	0.110
	**rs73594404**	GG	374 (91.0)	79 (84.0)	1.00 (reference)	
		GA	36 (8.8)	14 (14.9)	2.118 (1.011–4.440)	**0.047**
		AA	1 (0.2)	1 (1.1)	3.484 (0.128–95.079)	0.459
		AA + GA vs GG			2.164 (1.049–4.464)	**0.037**
		AA vs GA + GG			3.260 (0.121–87.981)	0.482
	**rs1059698**	AA	173 (42.1)	50 (53.2)	1.00 (reference)	
		CA	185 (45.0)	37 (39.4)	0.780 (0.469–1.295)	0.336
		CC	53 (12.9)	7 (7.4)	0.395 (0.161–0.971)	**0.043**
		CC + CA vs AA			0.660 (0.408–1.070)	0.092
		CC vs CA + AA			0.443 (0.185–1.058)	0.067
**Anemia**			Grade ≤ 0 N (%)	Grade > 0 N (%)		
	**rs10132552**	TT	141 (49.0)	110 (50.7)	1.00 (reference)	
		CT	129 (44.8)	79 (36.4)	0.764 (0.500–1.169)	0.215
		CC	14 (4.9)	23 (10.6)	2.653 (1.172–6.008)	**0.019**
		CC + CT vs TT			0.929 (0.618–1.398)	0.725
		CC vs CT + TT			3.001 (1.355–6.646)	**0.007**
	**rs73594404**	GG	263 (91.3)	190 (87.6)	1.00 (reference)	
		GA	25 (8.7)	25 (11.5)	2.109 (1.062–4.188)	**0.033**
		AA	0 (0)	2 (0.9)	NA	NA
		AA + GA vs GG			2.239 (1.138–4.405)	**0.020**
		AA vs GA + GG			NA	NA
**Myelosuppression**			Grade ≤ 2 N (%)	Grade > 2 N (%)		
	**rs1059698**	AA	161 (41.9)	62 (51.2)	1.00 (reference)	
		CA	172 (44.8)	50 (41.3)	0.810 (0.510–1.287)	0.373
		CC	51 (13.3)	9 (7.4)	0.407 (0.180–0.920)	**0.031**
		CC + CA vs AA			0.708 (0.456–1.098)	0.123
		CC vs CA + AA			0.449 (0.204–0.987)	**0.046**

Abbreviations: CI, confidence interval; OR, odds ratio.

*P* ^a^ values were calculated with adjustment for age, sex, BMI, smoking status, drinking status, histological type, clinical stage, T-staging, N-staging, Induction chemotherapy regimens, concurrent chemoradiotherapy regimen, irradiation dose.

*P* value < 0.05 is shown in bold.

### Multivariate Analysis of Selected SNPs as Prognostic Factors of Haematological Toxicities

#### Neutropaenia

Three SNPs were significantly associated with neutropaenia: rs2027701, rs73594404 and rs1059698. *LINC*-*ROR* rs2027701 showed an obvious trend towards a superior reaction with toxic effects in patients with one or two variant alleles compared with those with the wild-type genotype (OR = 0.503, 95%CI = 0.303–0.835, *P* = 0.008). As demonstrated, the *LINC-PINT* rs1059698 CC genotype was a protective factor (OR = 0.395, 95%CI = 0.161–0.971, P = 0.043), whereas *LINC-PINT* rs2293750 had no association with the risk of neutropaenia. In contrast to rs2027701 and rs1059698, rs73594404 *pR-lncRNA-1* had a weak correlation with increased risk of adverse reactions when patients possessed the GA genotype (OR = 2.118, 95%CI = 1.011–4.440, *P* = 0.047).

#### Anaemia

Patients with a minor A allele of *pR-lncRNA-1* rs73594404 had an increased risk of anaemia (OR = 2.109, 95%CI = 1.062–4.188, *P* = 0.033). The *MEG3* rs10132552 CC genotype correlated with a significantly inferior toxic reaction (OR = 2.653, 95%CI = 1.172–6.008, *P* = 0.019), which is an independent predictor for prognosis (CC vs CT + TT, OR = 3.001, 95%CI = 1.355–6.646, *P* = 0.007).

#### Myelosuppression

Statistical results indicated that only *LINC-PINT* rs1059698 polymorphisms had a correlation with CRT-induced myelosuppression. Although this SNP was not correlated with dermatitis and anaemia, we found that it had a significant association with not only the risk of neutropaenia (OR = 0.395, 95%CI = 0.161–0.971, *P* = 0.043), but also myelosuppression (OR = 0.407, 95%CI = 0.180–0.920, *P* = 0.031).

According to the multivariate logistic regression analyses, selected 8 SNPs did not interact with leukopaenia and thrombocytopenia in this study.

### Stratification Analysis of the Association between SNPs in lncRNA-p53 Regulatory Network Genes and Toxic Reactions

Supplementary Table S1 lists the relevancies between patient-related, tumour-related and therapy-related characteristics and the risk of toxicities generated by CRT. There was a significant dependency between gender, IC regimen, CRT regimen and multifarious toxicities. Female gender was an adverse factor for toxic reactions. Patients treated with TP, DP or DDP during CRT had a higher risk of anaemia compared with those treated with FP. Moreover, both age and BMI impacted individual risks. Patients with advanced T stages had a more than 1.5-fold greater myelosuppression risk compared with those in the early stages.

According to the above evidence, stratified analysis by adjusting for sex, IR regimen and CRT regimen was conducted to estimate the associations between the enrolled SNP polymorphisms and adverse reactions **(**Table 4 **)**. The rs10132552 CT genotype had an increased risk of leukopaenia, neutropaenia and myelosuppression in subgroups of the DP induction protocol. *pR-lncRNA-1* rs73594404 was another vital SNP that showed a strong relationships with toxicities in the IC regimen subgroup (OR = 3.394 and *P* = 0.015 for leukopaenia; OR = 3.540 and *P* = 0.036 for thrombocytopenia; OR = 3.054 and *P* = 0.022 for myelosuppression). Of significant interest, two SNPs, rs2027701 and rs1059698, had a weak correlation with oral mucositis in the subgroups of female (OR = 3.375 and *P* = 0.045) and IC regimen-DP (OR = 0.527 and *P* = 0.049), respectively.

**Table 4:** Stratification analysis of association between SNPs in lncRNA-p53 regulatory network genes and the toxic reactions in NPC patients.

Stratified factors	n	SNP	Toxic reactions	OR (95% CI)	*P*
IC regimen-TP	203	rs2027701	Dermatitis	4.721 (1.141–19.536)	**0.032**
		rs73594404	Leukopenia	3.394 (1.263–9.123)	**0.015**
			Myelosuppression	3.054 (1.178–7.922)	**0.022**
IC regimen-DP	200	rs1059698	Mucositis	0.527 (0.279–0.997)	**0.049**
		rs10132552	Leukopenia	4.300 (1.345–13.748)	**0.014**
			Neutropenia	5.462 (1.836–16.251)	**0.002**
		rs73594404	Thrombocytopenia	3.540 (1.089–11.500)	**0.036**
Sex-Female	131	rs2027701	Mucositis	3.375 (1.025–11.108)	**0.045**
		rs73594404	Thrombocytopenia	10.237 (2.530–41.429)	**0.001**
		rs10132552	Myelosuppression	4.135 (1.303–13.119)	**0.016**
CCRT regimen-TP	108	rs73594404	Leukopenia	3.784 (1.086–13.184)	**0.037**

Abbreviations: OR, odds ratio; CI, confidence interval;

*P* value < 0.05 is shown in bold.

**Figure 2:**
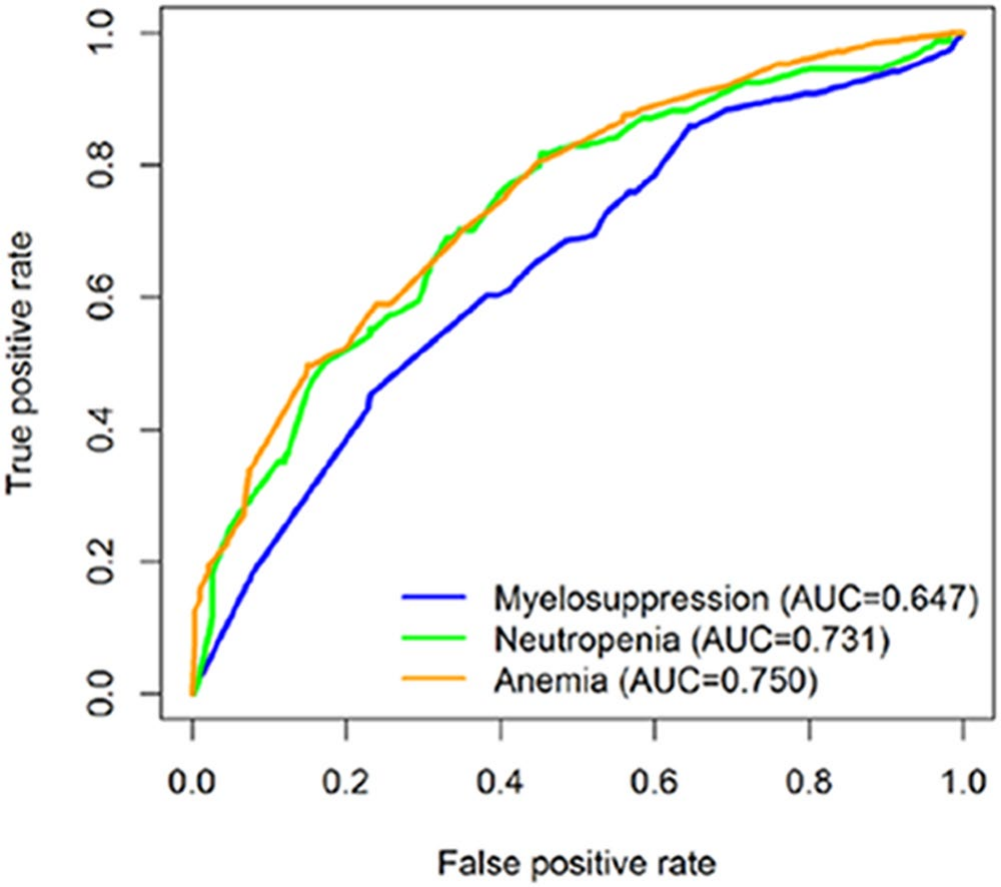
Analysis of receiver-operating characteristic curve to predict toxicities. ROC analysis showed the AUC of myelosuppression, neutropaenia and anaemia was 0.647, 0.731 and 0.750, respectively.

Hence, to further obtain the predictive power of lncRNAs after adjusting for clinical variables, a risk score model was built in accordance with the regression coefficients of variables to predict each patient’s risk of developing toxicities including neutropaenia, anaemia and myelosuppression (factors involved in P < 0.05). Using receiver operating characteristic (ROC) curve analysis, the prognostic power was evaluated **(**Fig. 2 **)**. The area under the curve (AUC) of neutropaenia, anaemia and myelosuppression was 0.731, 0.750 and 0.648, respectively, indicating good performance of the lncRNAs combined with clinical information for predicting toxicities in NPC patients.

### Interaction between Selected SNPs and the Efficacy of CRT 3 Months after Treatment

All positive results concerning the relationship between selected SNPs and the efficacy of CRT on the primary tumour and lymph nodes are listed in Table 5. Our results provide a plausible link between *LINC-ROR* rs2027701 polymorphisms and efficacy at the lymph node 3 months after CRT (OR = 2.266, 95%CI = 1.020–5.033, *P* = 0.045), while no significant difference was found on the primary tumour. In contrast, the *MEG3* rs10132552 CT genotype had a better response to treatments for the primary tumour compared with the TT genotype (OR = 0.261, 95%CI = 0.089–0.770, *P* = 0.015). With respect to the lymph node, rs10132552 polymorphisms are nonfunctional. We also analysed the connections between clinical factors and treatment efficacy; positive results are displayed in Supplementary Tables S2.

**Table 5:** Association between rs2027701 and rs10132552 and the efficacy of CRT at the primary tumor and lymph node 3 months after treatment in NPC patients

SNP		Primary tumor 3 months after treatment			*P* ^c^	Lymph node 3 months after treatment			*P* ^c^
		Genotype distribution (%)		OR (95% CI)		Genotype distribution		OR (95% CI)	
		Non-sensitive^a^	Sensitive^b^			Non-sensitive^a^	Sensitive^b^		
rs2027701	AA	8 (32.0)	125 (32.1)	1.00 (reference)		15 (24.2)	118 (33.5)	1.00 (reference)	
	GA	14 (56.0)	193 (49.6)	1.289 (0.467–3.558)	0.624	30 (48.4)	177 (50.3)	1.280 (0.643–2.549)	0.483
	GG	3 (12.0)	68 (17.5)	0.564 (0.131–2.426)	0.442	17 (27.4)	54 (15.3)	2.266 (1.020–5.033)	**0.045**
rs10132552	TT	18 (72.0)	187 (48.1)	1.00 (reference)		35 (56.5)	170 (48.3)	1.00 (reference)	
	CT	6 (24.0)	169 (43.4)	0.261 (0.089–0.770)	**0.015**	23 (37.1)	152 (43.2)	0.735 (0.398–1.358)	0.326
	CC	1 (4.0)	28 (7.2)	0.219 (0.024–1.990)	0.177	2(3.2)	27 (7.7)	0.331 (0.069–1.588)	0.167

^a^Complete response (CR) and partial response (PR).

^b^Stable disease (SD) and progressive disease (PD).

^c^Adjusted for age, sex, BMI, smoking status, drinking status, histological type, clinical stage, T-staging, N-staging, induction chemotherapy regimens, concurrent chemoradiotherapy regimen, irradiation dose for the association between SNPs and the efficacy of CRT at the primary tumor and lymph node 3 months after treatment.

Abbreviations: OR, odds ratio; CI, confidence interval.

*P* value < 0.05 is shown in bold.

## Discussion

In this study, we estimated the association of 8 SNPs in the lncRNA-p53 regulatory network of genes and the efficacy and toxic reactions in 505 NPC patients. To the best of our knowledge, this is the first study to demonstrate that 2 SNPs (*TUSC7* rs1829346 and rs36080650) were correlated with dermatitis, 3 SNPs (*LINC-ROR* rs2027701, *pR-lncRNA-1* rs73594404 and *LINC-PINT* rs1059698) were related to neutropaenia, 2 SNPs (*MEG3* rs10132552, *pR-lncRNA-1* rs73594404) and 1 SNP (*LINC-PINT* rs1059698) were associated with anaemia and myelosuppression in NPC patients, respectively. Of these, rs2027701 and rs10132552 were related to curative efficacy 3 months after treatment.

The regulation of the p53 tumour-suppressor pathway by lncRNAs, directly or indirectly, has been a hot topic of particularly intense interest. Non-coding mutations contributing to small changes in gene expression can have a large phenotypic impact on carcinoma, perhaps to an even greater degree than currently appreciated. As regulators, lncRNAs could affect p53 expression by influencing p53 mRNA stability or reducing its ability to recognize some of its binding sites, ultimately inhibiting p53 transcription^22,23^. lncRNAs also act as regulators that activate p53 directly by interacting with p53REs, regulating the gene expression of the p53 pathway at multiple levels and even establishing a regulatory feedback loop with p53. It is tempting to speculate that the interaction between p53 and lncRNAs has an impact on cell proliferation, cell cycle and cell apoptosis upon DNA damage, resulting in personalized differences in the toxic reactions and efficacy in NPC.

*TUSC7* was significantly induced in cells expressing wild-type p53, serving as a putative tumour suppressor by inhibiting cell growth, which plays a critical role in cancer prognosis, including oesophageal squamous cell carcinoma (ESCC)^24^, colorectal cancer^25^ and osteosarcoma^26^. Low expression of *TUSC7* was dramatically negatively correlated with the pathologic response to CRT and resulted in a poorer prognosis in cancers than the higher expression group^16^. Similarly, we found that rs1829346 and rs36080650 were significantly correlated with the risk of dermatitis under CRT. Patients carrying a homozygous mutation of these two SNPs were less resistant to grade 3–4 dermatitis. Unfortunately, the combination of the two SNPs did not increase risk. Accumulating studies have revealed that lncRNAs impact cellular functions through various mechanisms, most notably as a ‘sponge’ to titrate miRNAs^27,28^. The variation in the rs1829346 sequence may create a new binding site for miR-1304 **(**Supplementary Table S3 **)**, inhibiting the expression of *TUSC7*, regulating cell proliferation proteins and ultimately leading to a higher risk of CRT-induced dermatitis. For rs36080650, although there is no direct evidence to illustrate its function, the strong linkage disequilibrium (LD) with rs1829346 suggested that the regulation of gene expression is not mediated by this SNP but by a variant in rs1829346.

*LINC-ROR*, was first discovered in induced pluripotent stem cells (iPSCs); since it was discovered, the number of studies in this area have increased dramatically. *LINC-ROR* is not only a p53 effector but also a p53 regulator; its depletion would lead to the upregulation of genes involved in the p53 response to DNA damage-inducing agents and other stresses responses^17,29^. In our study, *LINC-ROR* rs2027701 showed an obvious trend towards a superior reaction with toxic effects in patients with one or two variant alleles, which may be explained by Zhang^21^, who found that *LINC-ROR* can significantly suppress p53 during DNA damage. Moreover, *LINC-ROR* suppression of the p53 pathway may account for patients who resist chemotherapy, thus playing a critical role in NPC. Our study found that rs2027701 polymorphisms were correlated with worse CRT efficacy at the lymph node.

*pR-lncRNA-1*, in a similar manner as *LINC-ROR*, is induced by p53 and modulates p53 transcriptional activity by forming an autoregulatory feedback loop with p53, enhancing p53 tumour suppressor activity and ultimately modulating the gene expression response to DNA damage^20^. *pR-lncRNA-1* rs73594404 has a strong connection to both neutropaenia and anaemia. Patients with the GA genotype, but not the AA genotype, have increased risk of CRT-induced toxicities. Although rs73594404 was identified in the intron region, it had enhancer activity and had its maximum signal strength in the region of transcription factor binding sites, suggesting that the rs73594404 mutant may influence gene expression by destroying the site or increasing the affinity of the transcription factor, leading to inactivation of tumour suppressor genes^30,31^. This links to poorer prognosis and additional studies will be required to confirm these observations.

*LINC-PINT* is a bona fide p53 transcriptional target that regulates cell proliferation by inducing cell apoptosis^19,32^. As shown in Table 2, the *LINC-PINT* rs1059698 CC genotype was a protective factor in neutropaenia and myelosuppression. Although studies on *LINC-PINT* are limited, using a database, we found that rs1059698 is located in the predicted active promoter flanking regions. The contribution of rare variants to asthma susceptibility is principally due to noncoding variants in sequences flanking the exons^33^. Similarly, Johnson^34^ revealed that genetic variation in noncoding sequences flanking the CYP3A locus was associated with the risk of breast cancer. Therefore, we speculated that the genetic polymorphism of rs1059698 would control promoter activity, inhibit *LINC-PINT* expression, and regulate a multitude of signalling pathways including the p53 network. Furthermore, rs1059698 is also located in the DNase I Hypersensitivity (DHS) cluster. Mutation of rs1059698 was strongly associated with transcription initiation activity, highlighting the role of rs1059698. However, further investigation is needed to delineate the precise mechanism.

**Figure 3:**
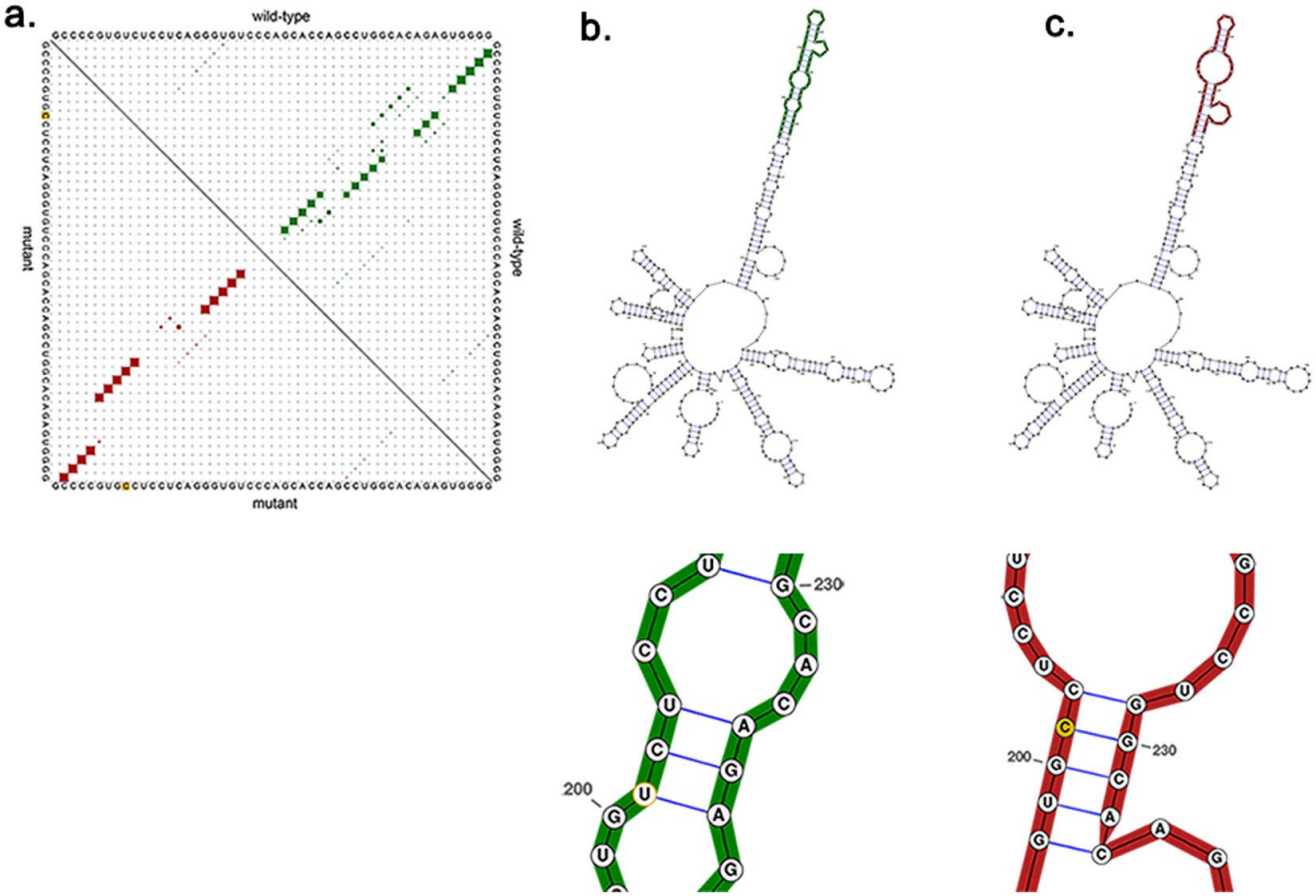
The predicting rs10132552 on *MEG3* secondary structure. (**a**) Base pair probabilities of the local region rs10132552. (**b**) The optimal secondary structure of global wild-type sequence. Minimum free energy −150.60 kcal/mol. (**c)** The optimal secondary structure of global mutant sequence. Minimum free energy −153.30 kcal/mol.

*MEG3* is a p53 regulator that is downregulated by MDM2, a well-known negative p53 regulator, thus controlling p53 stability and regulating downstream genes. *MEG3*, a tumour suppressor, has a great capacity for prognosis in many cancers. The *MEG3* rs7158663 AA genotype has significantly increased colorectal cancer risk, as revealed by Cao^35^. Analogously, we found that the *MEG3* rs10132552 CC genotype correlates with a significantly inferior toxic reaction; however, individuals with the CT genotype had a better response to treatments, suggesting that rs10132552 polymorphisms may impact the expression of *MEG3*, thus influencing p53 and subsequently suppressing cell proliferation or promoting cell apoptosis. Furthermore, functional genomic analyses were designed to provide a potential biological basis for the observed associations. Rs10132552 polymorphisms could create miRNA (mir-564, mir-650 and mir-602) binding sites on *MEG3*, disturbing the lncRNA-miRNA interaction, acting as competing endogenous RNAs (ceRNA) and thereby negatively regulating miRNA expression^36,37^. Interestingly, it is clear that mir-650, mir-602 and mir-564 played roles in cancers such as breast cancer and lung cancer^38–40^. Performing an *in silico* analysis, we found that when the T allele was substituted by the C allele, the structure of the transcript was changed **(**Fig. 3 **)**, leading to a minimum free energy (MFE) change from −150.60 kcal/mol to −153.30 kcal/mol, thus elucidating an important role for SNPs on RNA structure and supporting the idea that even SNPs can alter local RNA folding structure^41^. The altered gene expression and secondary structure may eventually result in different sensitivities of individuals to anaemia.

However, genetic polymorphisms are not the only signature of neutropaenia in NPC patients, and gemcitabine plus cisplatin was associated with increased risk of grade 3–4 haematological adverse events such as neutropaenia compared with fluorouracil plus cisplatin^42^, which could be supported by our data (40% had grade 3–4 neutropaenia in the GP group vs 7.6% in the PF group). Furthermore, we combined SNPs (rs2027701, rs73594404 and rs1059698) and clinical information to conduct ROC curves; the AUC of neutropaenia was 0.731, indicating good performance for predicting an adverse effect.

Some strengths of this study should be noted. First, this is the first study to explore the impact of SNPs on genes of the lncRNA-p53 regulatory network and the efficacy and toxic reactions in NPC patients. Second, a well-defined cohort of pathological diagnosed cases and strict inclusion criteria were used to avoid possible confounding factors that could hinder analysis. Finally, we obtained all of the essential clinical data from the included individuals. However, several limitations should not be ignored. First, our study is limited as a retrospective study at a single centre, thus selection bias could not be avoided. Second, the sample size seems to be too small for stratification analysis; therefore, statistical power may be limited. Finally, this pathway is complex-the five genes included in this analysis were insufficient and further study is needed.

In conclusion, we found six potential SNPs in five genes in the lncRNA-p53 regulatory network that are significantly associated with the toxicities and efficacy of CRT in 505 patients with NPC, thus providing new biomarkers that can predict therapeutic effect and acute toxic reactions. This study represents a significant step forward toward a better understanding of the importance of lncRNA-p53 in NPC.

## Methods

### Patient selection

This study consisted of 505 newly diagnosed NPC cases from the Affiliated Cancer Hospital of Xiangya School of Medicine, Central South University between 2014 and 2016. Peripheral blood specimens for genetic analysis were collected from each patient at the time of diagnosis. Patients were enrolled if they met the following criteria: (a) pathologically confirmed NPC; (b) Karnofsky score ≥70; (c) received intensity modulated radiation-therapy (IMRT) and concurrent chemoradiotherapy; and (d) patients without recurrence, metastasis and other malignancy. Patient demographics and clinical characteristics are shown in Table 1. This study was performed with the approval of the Independent Ethical Committee of the Institute of Clinical Pharmacology, Central South University (CTXY-140007–2). At recruitment, written informed consent was obtained from all participants involved in this study. All experiments methods were performed in accordance with the relevant guidelines and regulations.

### Efficacy regimen

All patients were treated with IMRT with the median total radiation dose of 71.34 Gy. The induction chemotherapy and concurrent chemotherapy were all performed with platinum-based chemotherapy regimens, including DP, docetaxel with cisplatin/ nedaplatin; FP, fluorouracil with cisplatin/nedaplatin; TP, paclitaxel with cisplatin/nedaplatin; GP, gemcitabine with cisplatin/ nedaplatin; DDP, cisplatin alone; NDP, nedaplatin alone.

### SNP selection and genotyping assays

We selected the SNPs for *TUSC7, LINC-ROR, pR-lncRNA-1, LINC-PINT* and *MEG3* by using databases including ENCODE, lncRNASNP, Hapmap and Ensembl to analyse their potential function. The SNPs were selected according to the following criteria: (1) with a minor allele frequency (MAF) ≥0.05 in a Southern Han Chinese population; (2) located in the promoter, miRNA binding site and other functional region; (3) SNPs have not been study before, not only NPC, but also other carcinomas. Genomic DNA was extracted from lymphocytes using the QIAamp DNA Blood Mini Kit (Qiagen, Valencia, CA) according to the manufacturer’s instructions, and stored at -80 °C until use. The DNA purity and concentration were determined by spectrophotometric measurement of absorbance at 260 nm and 280 nm. The candidate SNPs were genotyped using the Sequenom MassARRAY iPLEX platform (Sequenom, Inc., San Diego, CA, USA). The detection rate of all SNPs was greater than 98%.

### Evaluations of toxic reactions

Acute CRT-induced toxic reactions including dermatitis, mucositis, leukopaenia, myelosuppression, neutropaenia, anaemia and thrombocytopenia were recorded and evaluated according to the Common Terminology Criteria for Adverse Events (CTCAE 3.0). We defined grade 0–2 as mild toxic reactions and grade 3–4 as severe side effects, except for anaemia and thrombocytopenia, which have a lower incidence rate, and we chose 0 as the cutoff target. All acute toxic reactions were observed once a week from the first day to the end of treatment. RECIST (Response Evaluation Criteria in Solid Tumours) was used to evaluate efficacy three months after treatment.

### Statistical analysis

Deviations from Hardy-Weinberg equilibrium were calculated using χ2 analysis. By computing the odds ratio (OR) and the corresponding 95% confidence interval (CI), and continuous variables such as age and BMI were switched to binary variables, multivariate logistic regression was performed to determine the association of each SNP of *TUSC7, LINC-ROR, pR-lncRNA-1, LINC-PINT* and *MEG3* with the CRT efficacy and toxic reactions, with adjustments for age, sex, clinical stage, treatment modality and other clinical factors. In addition, stratification analyses were performed to characterize the associations between SNPs and toxic reactions in some subgroups when the corresponding clinical factors had an impact on toxic effects. We used receiver operating characteristic (ROC) curve analysis to evaluate the prognostic power of SNPs by comparing area under the curve (AUC) for each ROC when the P value < 0.05 was considered statistically significant. All statistical analyses were performed with SPSS19.0 and the R package. We also used lncRNASNP (http://bioinfo.life.hust.edu.cn/lncRNASNP/, accessed 30 January 2017) and RNASNP (http://rth.dk/resources/rnasnp/, accessed 30 January 2017) to predict the folding structure variants of genes due to SNP genotypes and the gain and loss of function of miRNA-lncRNA interactions through SNP polymorphisms, respectively.

### Data Availability

All data generated or analyzed during this study are included in this published article (and its Supplementary Information files). But the raw data used for the analysis and genotyping data for each patient is private.

## Electronic supplementary material

Supplementary Table S1-S3
