# Author Correction: Genetic polymorphisms of lncRNA-p53 regulatory network genes are associated with concurrent chemoradiotherapy toxicities and efficacy in nasopharyngeal carcinoma patients

**DOI:** 10.1038/s41598-017-16511-1

**Published:** 2017-11-22

**Authors:** Youhong Wang, Zhen Guo, Yu Zhao, Yi Jin, Liang An, Bin Wu, Zhaoqian Liu, Xiaoping Chen, Xiang Chen, Honghao Zhou, Hui Wang, Wei Zhang

**Affiliations:** 1 Department of Clinical Pharmacology, Xiangya Hospital, Central South University and Institute of Clinical Pharmacology, Central South University; Hunan Key Laboratory of Pharmacogenetics, Changsha, 410008 P.R. China; 2 Department of Radiation Oncology, Hunan Provincial Tumor Hospital & Affiliated Tumor Hospital of Xiangya Medical School, Central South University; Hunan Key Laboratory of Translational Radiation Oncology, ChangSha, 410013 P.R. China; 3 Department of Dermatology, Xiangya Hospital, Central South University; Hunan Key Laboratory of Skin Cancer and Psoriasis, Changsha, Hunan 410008 China

*Scientific Reports* **7**:8320; doi:10.1038/s41598-017-08890-2; Article published online 16 August 2017

The original PDF version of this Article contained an error in the order of corresponding authors. This has now been corrected in the PDF version of the Article.

