# Supplementary material for: Genetic polymorphisms of lncRNA-p53 regulatory network genes are associated with concurrent chemoradiotherapy toxicities and efficacy in nasopharyngeal carcinoma patients
Source: Sci Rep. 2017 Aug 16;7:8320. doi: 10.1038/s41598-017-08890-2 (PMC5559481; doi:10.1038/s41598-017-08890-2)
Supplement: Supplementary file 1 — Supplementary Table S1-S3 [file 41598_2017_8890_MOESM1_ESM.pdf]

Genetic polymorphisms of lncRNA-p53 regulatory network genes are associated with the concurrent chemoradiotherapy toxicities and efficacy in nasopharyngeal carcinoma patients

Youhong Wang[1], Zhen Guo[1], Yu Zhao[2], Yi Jin[2], Liang An[1], Bin Wu[1], Zhaoqian Liu[1], Xiaoping Chen[1], Xiang Chen[3], Honghao Zhou[1], Hui Wang[2,*] , Wei Zhang[1,*]

* To whom correspondence should be addressed. Wei Zhang, Department of Clinical Pharmacology, Xiangya Hospital, Central South University, 110 Xiang Ya Road, Changsha, Hunan 410078, P.R. China. Email: yjsd2003@163.com.

Supplementary Table S1 Associations Between Patient-Related, Tumor-Related, and Therapy-Related Characteristics and Concurrent Chemoradiotherapy-Induced Toxicity

		Dermatitis		Mucositis		Leukopenia		Neutropenia		Anemia		Thrombocytopenia		Myelosuppression	
Parameter	No.	OR (95%CI)	P[a]	OR (95%CI)	P[a]	OR (95%CI)	P[a]	OR (95%CI)	P[a]	OR (95%CI)	P[a]	OR (95%CI)	P[a]	OR (95%CI)	P[a]
Sex															
Male	374	1.000		1.000		1.000		1.000		1.000		1.000		1.000	
Female	131	0.641(0.246-1.532)	0.296	1.240(0.693-2.221)	0.468	2,236(1.281-3.901)	**0.005**	1.830(1.078-3.109)	**0.025**	3.776(2.385-5.978)	**0.000**	0.708(0.366-1.369)	0.304	1.808(1.115-2.931)	**0.016**
Age, y															
< 47	229	1.000		1.000		1.000		1.000		1.000		1.000		1.000	
≥ 47	276	1.136(0.621-2.079)	0.679	0.818(0.539-1.242)	0.346	1.008(0.586-1.733)	0.978	1.251(0.765-2.044)	0.372	2.698(1.795-4.056)	**0.000**	2.370(1.456-3.857)	0.001	1.190(0.762-1.859)	0.445
BMI															
< 18.5	30	1.000		1.000		1.000		1.000		1.000		1.000		1.000	
18.5 ~ 24	274	0.832(0.263-2.638)	0.755	0.575(0.248-1.332)	0.197	0.673(0.244-1.860)	0.445	0.912(0.330-2.523)	0.860	0.648(0.281-1.492)	0.308	0.936(0.350-2.506)	0.895	1.083(0.420-2.790)	0.869
≥ 24	201	0.585(0.171-2.000)	0.393	0.711(0.298-1.698)	0.442	0.519(0.176-1.527)	0.234	0.745(0.260-2.135)	0.584	0.287(0.120-0.685)	**0.005**	0.706(0.251-1.990)	0.510	0.903(0.338-2.408)	0.838
Smoking status															
Nonsmoker	258	1.000		1.000		1.000		1.000		1.000		1.000		1.000	
Smoker	247	1.560(0.867-2.807)	0.138	1.033(0.613-1.741)	0.904	1.244(0.597-2.591)	0.560	0.845(0.454-1.573)	0.595	0.833(0.504-1.377)	0.475	0.878(0.5-1.542)	0.650	0.765(0.437-1.342)	0.351
Drinking status															
Nondrinker	415	1.000		1.000		1.000		1.000		1.000		1.000		1.000	
Drinker	90	0.547(0.228-1.313)	0.177	1.106(0.625-1.956)	0.730	0.959(0.431-2.135)	0.918	0.763(0.369-1.580)	0.467	1.385(0.802-2.394)	0.243	0.894(0.476-1.678)	0.727	1.080(0.578-2.019)	0.810
Histological type															
WHO type II	214	1.000		1.000		1.000		1.000		1.000		1.000		1.000	
WHO type III	291	1.111(0.607-2.032)	0.734	0.789(0.521-1.197)	0.265	1.559(0.901-2.700)	0.113	1.419(0.856-2.351)	0.174	1.065(0.711-1.595)	0.761	1.000(0.627-1.596)	1.000	1.444(0.924-2.258)	0.107
Clinical stage															
I-II	50	1.000		1.000		1.000		1.000		1.000		1.000		1.000	
III-IV	455	1.175(2.247-5.588)	0.839	2.212(0.424-3.464)	0.719	8.363(1.658-42.18)	**0.010**	1.157(0.362-3.695)	0.806	0.865(0.313-2.392)	0.780	1.818(0.576-5.736)	0.308	1.754(0.579-5.318)	0.321

T-staging															
T1-T2	246	1.000		1.000		1.000		1.000		1.000		1.000		1.000	
T3-T4	259	1.112(0.583-2.120)	0.747	1.210(0.769-1.903)	0.410	1.189(0.663-2.132)	0.562	1.516(0.927-2.478)	0.097	1.434(0.960-2.142)	0.078	1.070(0.640-1.787)	0.797	1.599(1.025-2.494)	**0.039**
N-staging															
N0-N1	93	1.000		1.000		1.000		1.000		1.000		1.000		1.000	
N2-N3	412	1.222(0.404-3.693)	0.723	0.843(0.402-1.771)	0.653	0.452(0.202-1.011)	0.053	0.683(0.304-1.534)	0.356	1.493(0.719-3.098)	0.282	0.641(0.297-1.382)	0.256	0.695(0.328-1.471)	0.341
IC regimen															
DP	200	1.000		1.000		1.000		1.000		1.000		1.000		1.000	
FP	92	1.070(0.262-4.364)	0.925	0.301(0.094-0.960)	**0.042**	5.138(1.121-23.54)	**0.035**	0.368(0.074-1.832)	0.222	0.863(0.306-2.432)	0.780	3.636(1.125-11.82)	**0.031**	1.429(0.412-4.951)	0.574
TP	203	0.869(0.330-2.292)	0.777	0.356(0.185-0.687)	**0.002**	2.097(0.825-5.330)	0.120	1.722(0.784-3.783)	0.176	1.198(0.643-2.235)	0.569	0.741(0.368-1.495)	0.403	1.993(0.993-4.000)	0.052
GP	10	0.000	0．999	0.314(0.083-2.226)	0.430	13.947(2.51-77.28)	**0.003**	4.156(0.970-17.805)	0.055	3.109(0.657-14.716)	0.153	6.566(1.611-26.77)	**0.009**	6.672(1.600-27.813)	**0.009**
CRT regimen															
FP	85	1.000		1.000		1.000		1.000		1.000		1.000		1.000	
TP	108	0.954(0.217-4.186)	0.950	1.521(0.453-5.102)	0.497	4.936(1.062-22.95)	**0.042**	1.580(0.361-6.922)	0.544	3.829(1.978-7.411)	**0.000**	4.438(1.185-16.62)	**0.027**	3.125(0.898-10.866)	0.073
DDP	83	1.050(0.284-3.887)	0.942	0.696(0.225-2.154)	0.529	0.844(0.198-3.590)	0.818	0.683(0.156-2.979)	0.612	3.182(1.601-6.326)	**0.001**	2.273(0.730-7.074)	0.156	1.079(0.327-3.559)	0.901
NDP	172	0.479(0.113-2.042)	0.320	0.573(0.176-1.862)	0.355	2.340(0.514-10.65)	0.272	0.538(0.121-2.401)	0.417	0.966(0.523-1.786)	0.913	4.089(1.171-14.28)	**0.027**	1.074(0.309-3.737)	0.911
DP	57	0.607(0.112-3.285)	0.563	0.293(0.077-1.113)	0.071	5.888(1.064-32.58)	**0.042**	1.295(0.255-6.588)	0.756	2.315(1.083-4.948)	**0.030**	1.110(0.236-5.230)	0.895	2.607(0.648-10.490)	0.177
pGTVnx															
<71.00Gy	261	1.000		1.000		1.000		1.000		1.000		1.000		1.000	
≥71.00Gy	234	0.710(0.364-1.388)	0.317	1.034(0.641-1.669)	0.891	0.713(0.392-1.297)	0.268	1.489(0.877-2.528)	0.141	1.170(0.745-1.837)	0.496	0.752(0.439-1.290)	0.301	1.096(0.670-1.791)	0.716

Abbreviations: CI, confidence interval; OR, odds ratio; BMI, Body Mass Index; IC regimen, Induction chemotherapy regimens; CRT regimen, concurrent chemoradiotherapy regimen; pGTVnx, irradiation dose.

P_a values were calculated with adjustment for age, sex, BMI, smoking status, drinking status, histological type, clinical stage, T-staging, N-staging, Induction chemotherapy regimens, concurrent chemoradiotherapy regimen, irradiation dose.

P<0.05 were shown in bold.

Supplementary Table S2 The associations between clinical factors and efficacy of 3 months after treatment on primary tumor and lymph node

Parameter		No.	Primary tumor 3 months after treatment		Lymph node 3 months after treatment	
			OR (95%CI)	*P*	OR (95%CI)	*P*
T-staging						
	T1-T2	246	1.00 (reference)		1.00 (reference)	
	T3-T4	259	0.351 (0.140-0.882)	**0.026**	0.557 (0.297-1.045)	0.068
IC regimen						
	DP	200	1.00 (reference)		1.00 (reference)	
	GP	10	0.146 (0.025-0.871)	**0.035**	1.064 (0.184-6.148)	0.994
	TP	203	1.424 (0.498-4.073)	0.510	5.423 (1.997-14.727)	**0.001**

Abbreviations: CI, confidence interval; OR, odds ratio; IC regimen, Induction chemotherapy regimens;

P<0.05 were shown in bold.

Supplementary Table S3 SNP causes miRNA : lncRNA Gain or Loss

<table>
<tr><th>Gene</th><th>SNP</th><th>miRNA ID</th><th></th><th>SNP causes miRNA : lncRNA Gain/Loss</th><th>Literature</th></tr>
<tr><td>MEG3</td><td>rs10132552</td><td>hsa-miR-564</td><td>Gain</td><td>miRNA: 3' cgGAC-GACUGUGGCACGGa 5'
:|| | :| |||||||
lncRNA: 5' ccTTGACCAGCCCCGTGCCt 3'</td><td>MiR-564 inhibits proliferation and invasion in breast cancer[1], lung cancer[2], glioblastoma cells[3].</td></tr>
<tr><td></td><td></td><td>hsa-miR-650</td><td>Gain</td><td>miRNA: 3' caGGACU-CUCGCG--ACGGAGGa 5'
|:||| ||| | |||||||
lncRNA: 5' ccCTTGACCAGCCCCGTGCCTCCt 3'</td><td>Higher expression of miR-650 is associated with a favorable CLL prognosis and influences the proliferation capacity of B cells[4].</td></tr>
<tr><td></td><td></td><td>hsa-miR-602</td><td>Loss</td><td>miRNA: 3' ccCGGCGUCGACAG--C-GGGCACAg 5'
| :|| :|| | | ||||||x
lncRNA: 5' ggGGTGCCCTTGACCAGCCCCGTGTc 3'</td><td>MiR-602 plays a pro-carcinogenic role in HBV-related hepatocarcinogenesis[5].</td></tr>
<tr><td>TUSC7</td><td>rs1829346</td><td>hsa-miR-1304</td><td>Gain</td><td>miRNA: 3' ccccAAGCUCCGAUGUCACUCu 5'
||||| ||||||||
lncRNA: 5' caaaTTCGA----ACAGTGAGc 3'</td><td>MiR-1304 suppresses human non-small cell lung cancer cell growth[6].</td></tr>
<tr><td>LINC-PINT</td><td>rs1059698</td><td>hsa-miR-646</td><td>Gain</td><td>miRNA: 3' cgGAGUCU-CCGUCGACGAa 5'
|| ||: | |||||||
lncRNA: 5' agCTAAGGTGAAAGCTGCTa 3'</td><td>MiR-646 correlated with tumor metastasis in clear cell renal carcinoma[7].</td></tr>
</table>

References

1. Mutlu, M. *et al*. miR-564 acts as a dual inhibitor of PI3K and MAPK signaling networks and inhibits proliferation and invasion in breast cancer. *Sci Rep* **6**, 32541 (2016).

2. Yang, B. *et al*. MiR-564 functions as a tumor suppressor in human lung cancer by targeting ZIC3. *Biochem Biophys Res Commun* **467**, 690-696 (2015).

3. Jiang, C. *et al*. MicroRNA-564 is downregulated in glioblastoma and inhibited proliferation and invasion of glioblastoma cells by targeting TGF-β1. *Oncotarget* **7**,56200-56208 (2016).

4. Mraz, M. *et al*. MicroRNA-650 expression is influenced by immunoglobulin gene rearrangement and affects the biology of chronic lymphocytic leukemia. *Blood* **119,** 2110-2113 (2012).

5. Yang, L. *et al*. MicroRNA-602 regulating tumor suppressive gene RASSF1A is overexpressed in hepatitis B virus-infected liver and hepatocellular carcinoma. *Cancer Biol Ther* **9**, 803-808 (2010).

6. Li, C. G. *et al*. MicroRNA-1304 suppresses human non-small cell lung cancer cell growth in vitro by targeting heme oxygenase-1. *Acta Pharmacol Sin* **38,** 110-119 (2017).

7. Li, W. *et al*. Downregulated miR-646 in clear cell renal carcinoma correlated with tumour metastasis by targeting the nin one binding protein (NOB1). *Br J Cancer* **111**, 1188-1200 (2014).
